# Infective Endocarditis on Aortic Valve: From Diagnosis to Cardiac Surgical Intervention—Narrative Review

**DOI:** 10.3390/jcm15166463

**Published:** 2026-08-20

**Authors:** Francesco Loreni, Federico Fortuni, Alessandro Affronti, Romina Pantanella, Simone Perticoni, Davide Di Lazzaro, Antonio Nenna, Raffaele Barbato, Ciro Mastroianni, Mario Lusini, Massimo Chello, Erberto Carluccio, Marcello Bergonzini

**Affiliations:** 1 Unit of Cardiac Surgery, Department of Medicine, Fondazione Policlinico Universitario Campus Bio-Medico, 00128 Rome, Italy; 2 Unit of Cardiac Surgery, Hospital Santa Maria Della Misericordia, 06129 Perugia, Italy; 3 Cardiology and Cardiovascular Pathophysiology, Santa Maria Della Misericordia Hospital, University of Perugia, 06123 Perugia, Italy; 4 Unit of Cardiac Surgery, Azienda Ospedaliera Maggiore della Carità, 28100 Novara, Italy; antonio.nnn@hotmail.it

**Keywords:** aortic, valve, endocarditis, aortic valve, cardiac surgery, surgery, surgical procedure, diagnosis, cardiology, innovation

## Abstract

Infective endocarditis (IE) continues to represent a major challenge for global health systems. In 2019, its annual incidence was estimated at 13.8 cases per 100,000 individuals, contributing to approximately 66,300 deaths worldwide. Due to its high morbidity and mortality rates, enhancing preventive measures has become a priority in both clinical practice and ongoing research efforts. Since the publication of the 2015 ESC Guidelines for the management of IE, several pivotal studies have emerged, prompting a re-evaluation and potential update of the existing recommendations. One growing concern is the increasing antibiotic resistance among oral streptococci, particularly to macrolides such as azithromycin and clarithromycin, which now show higher resistance levels than penicillin. Changes in national antibiotic stewardship programs may have inadvertently contributed to a rise in IE incidence, in part due to altered prophylactic practices. At the same time, advances in diagnostic modalities—including more widespread and targeted use of echocardiography in patients with positive blood cultures for organisms like Enterococcus faecalis, Staphylococcus aureus, and various streptococci—have likely improved detection rates. Additionally, innovations in imaging, particularly computed tomography (CT) and nuclear medicine techniques, have enhanced the diagnosis of IE, especially among patients with prosthetic heart valves or implantable cardiac devices. This has allowed for better characterization of patient populations, aiding in the refinement of diagnostic criteria and therapeutic approaches. Furthermore, updated antibiotic treatment protocols, informed by EUCAST’s antimicrobial susceptibility data, have helped tailor antimicrobial regimens to current resistance trends. The combination of improved diagnostic sensitivity and evolving microbial resistance patterns has also led to an increased number of patients being considered for cardiac surgery as part of their treatment pathway. This review seeks to synthesize the latest findings and guideline revisions, offering an integrated overview of recent progress in the diagnosis, medical treatment, and surgical management of infective endocarditis. It will also explore current therapeutic strategies and operative indications in light of the most recent evidence.

## 1. Introduction

Infective endocarditis (IE) is now recognized as a significant public health concern. In 2019, the estimated annual incidence was 13.8 cases per 100,000 individuals, with 66,300 deaths worldwide attributed to IE. Due to the high morbidity (1723.59 disability-adjusted life years) and mortality (0.87 deaths per 100,000 population), research has primarily focused on identifying the most effective preventive strategies. Since the publication of the previous ESC guidelines for the management of IE in 2015, new and relevant data have emerged, making it necessary to update the recommendations with the release of the 2023 ESC guidelines. Specifically: An increase in the population at risk of IE and new data regarding its presentation in different clinical settings. Rising antimicrobial resistance among oral streptococci, with resistance rates to azithromycin and clarithromycin exceeding those observed for penicillin [1]. Uncertainty about whether changes in national guidelines on antibiotic prophylaxis have influenced the incidence of IE. Broader use of advanced diagnostic techniques (such as echocardiography, CT, and nuclear imaging), likely contributing to the increased detection of IE cases, especially among patients with prosthetic valves or implantable cardiac devices. To update the recommendations for the diagnosis and treatment of IE, the following elements were taken into account:

- Contemporary data on the characteristics of patients affected by IE;
- Antimicrobial susceptibility profiles of different pathogens based on the clinical breakpoints defined by EUCAST (European Committee on Antimicrobial Susceptibility Testing);
- Recommendations for outpatient parenteral antibiotic therapy (OPAT) and partial oral antibiotic treatment, based on evidence from the POET (Partial Oral Treatment of Endocarditis) randomized trial and other studies.

These considerations have guided the revision of diagnostic and therapeutic strategies to improve the management of infective endocarditis [1,2,3,4,5,6,7,8,9,10,11,12,13,14,15,16,17,18,19,20,21,22,23,24,25,26,27,28,29,30].

## 2. Materials and Methods

A comprehensive literature search was performed using PubMed/MEDLINE, Scopus, and Web of Science to identify relevant studies on aortic valve infective endocarditis published between January 2000 and March 2025. The search included the terms infective endocarditis, aortic valve, echocardiography, cardiac CT, MRI, antimicrobial therapy, valve repair, valve replacement, and surgery. Priority was given to international guidelines, systematic reviews, meta-analyses, randomized trials, and high-quality observational studies. Only English-language studies involving adult patients were included. References were selected according to their methodological quality, clinical relevance, and contribution to the current understanding of the diagnosis, imaging, medical treatment, surgical management, and outcomes of aortic valve infective endocarditis.

## 3. Diagnosis of IE

The clinical presentation of infective endocarditis (IE) is highly heterogeneous, ranging from acute sepsis to subacute or chronic disease with low-grade fever or nonspecific symptoms. Because IE may mimic rheumatologic, neurologic, autoimmune, or neoplastic disorders, diagnosis is often delayed. Therefore, IE should be suspected in any patient with fever or sepsis of unknown origin, particularly in the presence of positive blood cultures without an identifiable infectious source or in patients with predisposing conditions such as prosthetic valves, congenital heart disease, or previous IE. Clinical evaluation should include assessment of cardiac and non-cardiac risk factors, potential portals of infection, and a thorough physical examination. However, classic clinical signs have limited sensitivity and specificity. According to the EURO-ENDO registry, the most common manifestations are fever (77.7%), cardiac murmurs (64.5%), and heart failure (27.2%), while embolic events and conduction disturbances occur in approximately one-quarter and one-tenth of patients, respectively. Peripheral vascular and immunologic manifestations remain uncommon but may still be observed, particularly in severe *Staphylococcus aureus* infections or subacute streptococcal IE. Elderly and immunocompromised patients frequently present with atypical manifestations, requiring a high index of clinical suspicion and early diagnostic evaluation [31,32,33,34,35].

### 3.1. Infective Endocarditis with Positive Blood Cultures

Blood cultures remain the cornerstone of IE diagnosis, allowing pathogen identification and antimicrobial susceptibility testing. Current recommendations advise obtaining at least three sets of peripheral blood cultures (10 mL each), approximately 30 min apart, before initiating antibiotic therapy, using strict aseptic technique and both aerobic and anaerobic incubation. Because bacteremia in IE is continuous, blood sampling does not need to coincide with fever spikes, and all cultures are expected to be positive. Consequently, an isolated positive blood culture should be interpreted cautiously. Close communication with the microbiology laboratory is essential. Automated blood culture systems, Gram staining, and MALDI-TOF mass spectrometry enable rapid pathogen identification, whereas antimicrobial therapy should ultimately be guided by standardized minimum inhibitory concentration (MIC) testing, which remains the reference method for susceptibility assessment [36,37,38,39].

### 3.2. Blood Culture-Negative Infective Endocarditis

**Figure 1:**
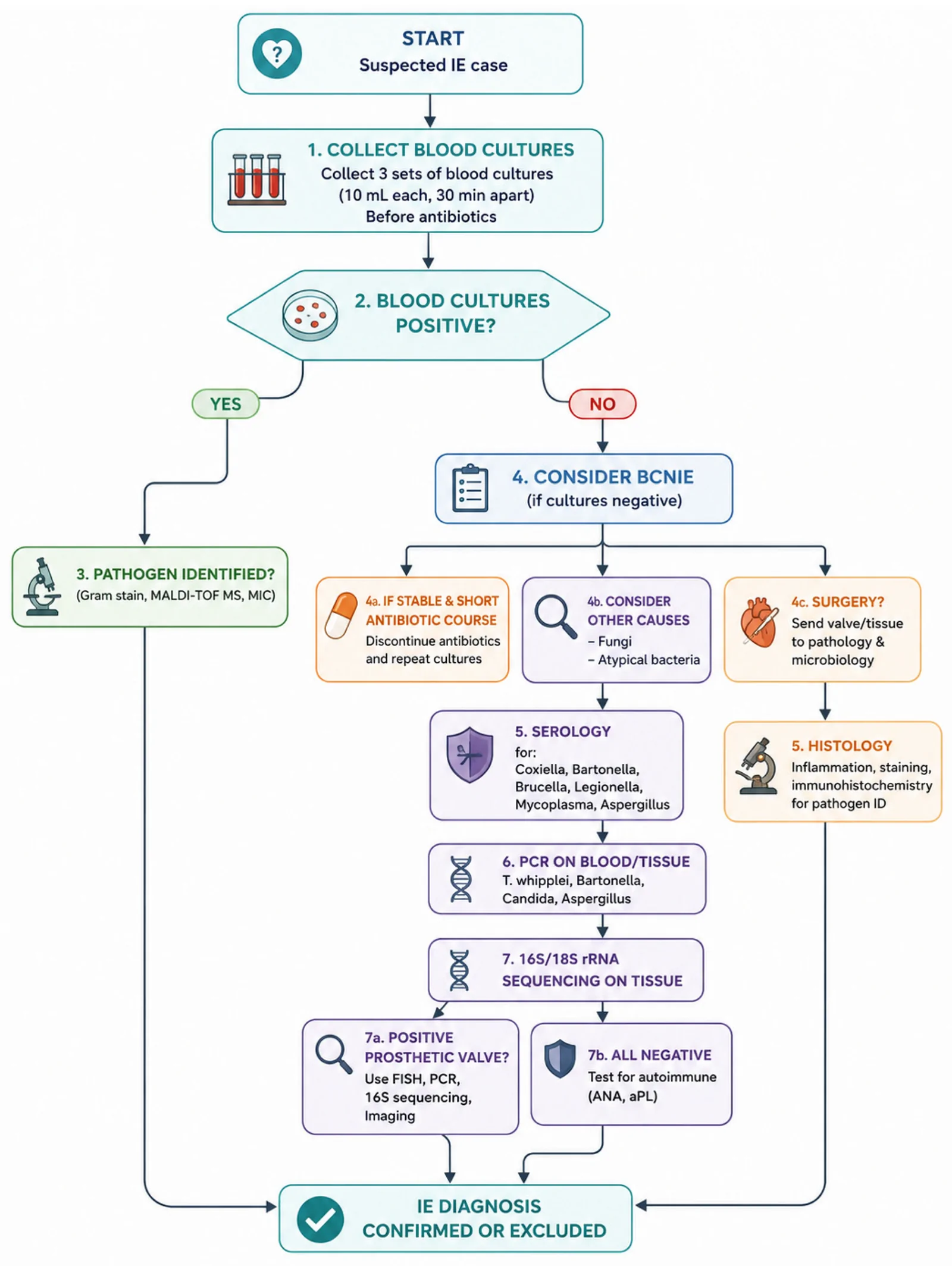
Diagnostic flow chart.

Blood culture-negative infective endocarditis (BCNIE) most commonly results from previous antibiotic exposure or infection with fastidious or intracellular microorganisms. In clinically stable patients who have recently received antibiotics, temporary discontinuation of antimicrobial therapy followed by repeat blood cultures may improve diagnostic yield. When BCNIE is suspected, the diagnostic work-up should include serological testing for *Coxiella burnetii*, *Bartonella* spp., *Brucella* spp., *Legionella pneumophila*, *Mycoplasma pneumoniae*, and *Aspergillus* spp., together with PCR assays on blood or tissue for *Tropheryma whipplei*, *Bartonella* spp., *Candida* spp., and *Aspergillus* spp. Molecular techniques, including 16S/18S rRNA sequencing, significantly improve pathogen detection, particularly in prosthetic valve endocarditis, where they may be combined with fluorescence in situ hybridization (FISH) and molecular imaging. Emerging microbial cell-free DNA sequencing may further enhance future diagnostic accuracy. If microbiological investigations remain negative, non-infectious causes of endocarditis, including autoimmune disorders, should be considered through appropriate immunological testing. Nevertheless, histopathological examination of excised valve tissue or embolic material remains the diagnostic gold standard. Valve specimens should be submitted simultaneously for pathology and microbiology, where histology, special stains, immunohistochemistry, and molecular analyses not only identify the causative pathogen but also help distinguish infectious from non-infectious forms of endocarditis [40,41,42,43] (Figure 1).

### 3.3. Multimodality Imaging

**Figure 2:**
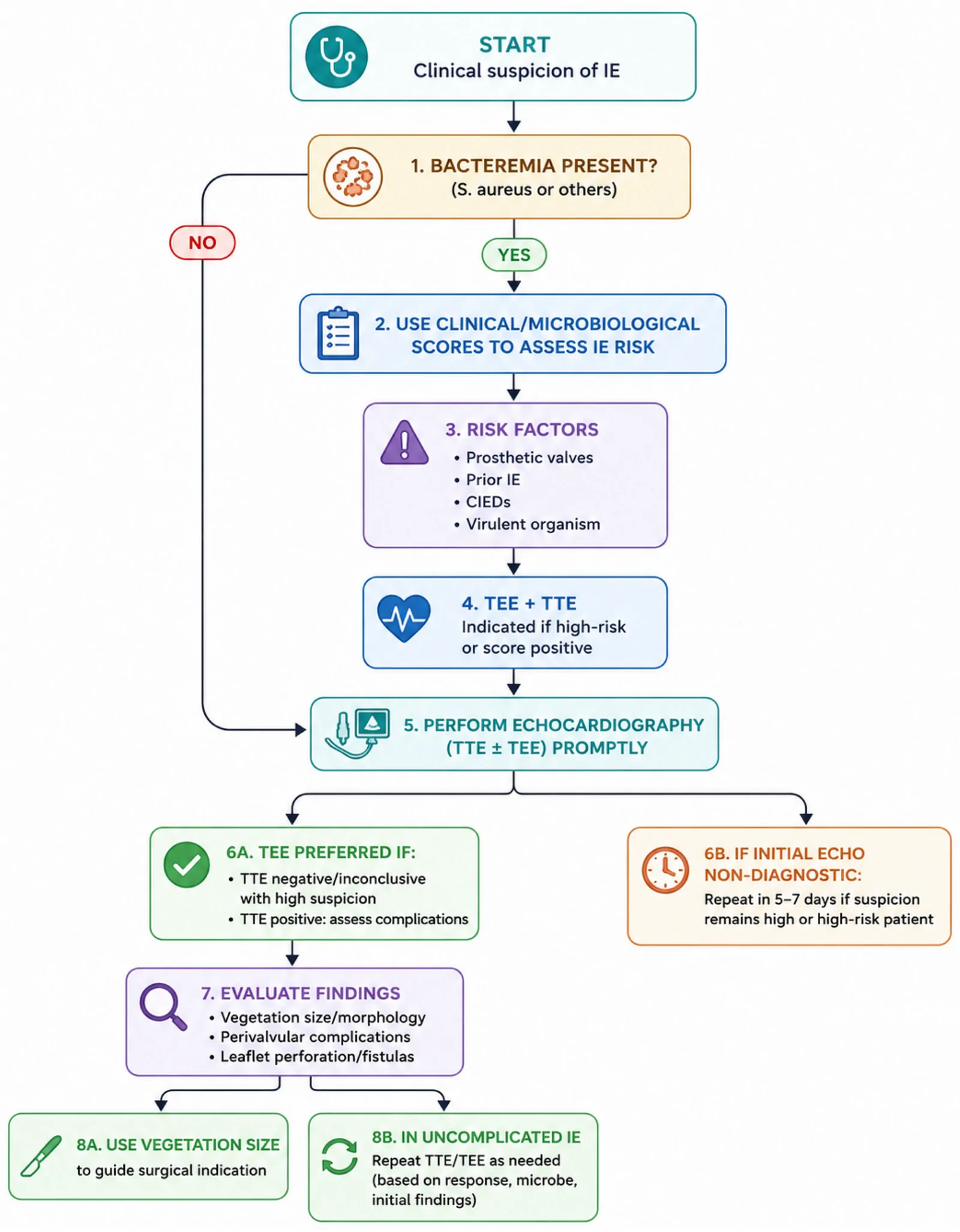
Echocardiography TEE or TTE flow chart.

**Figure 3:**
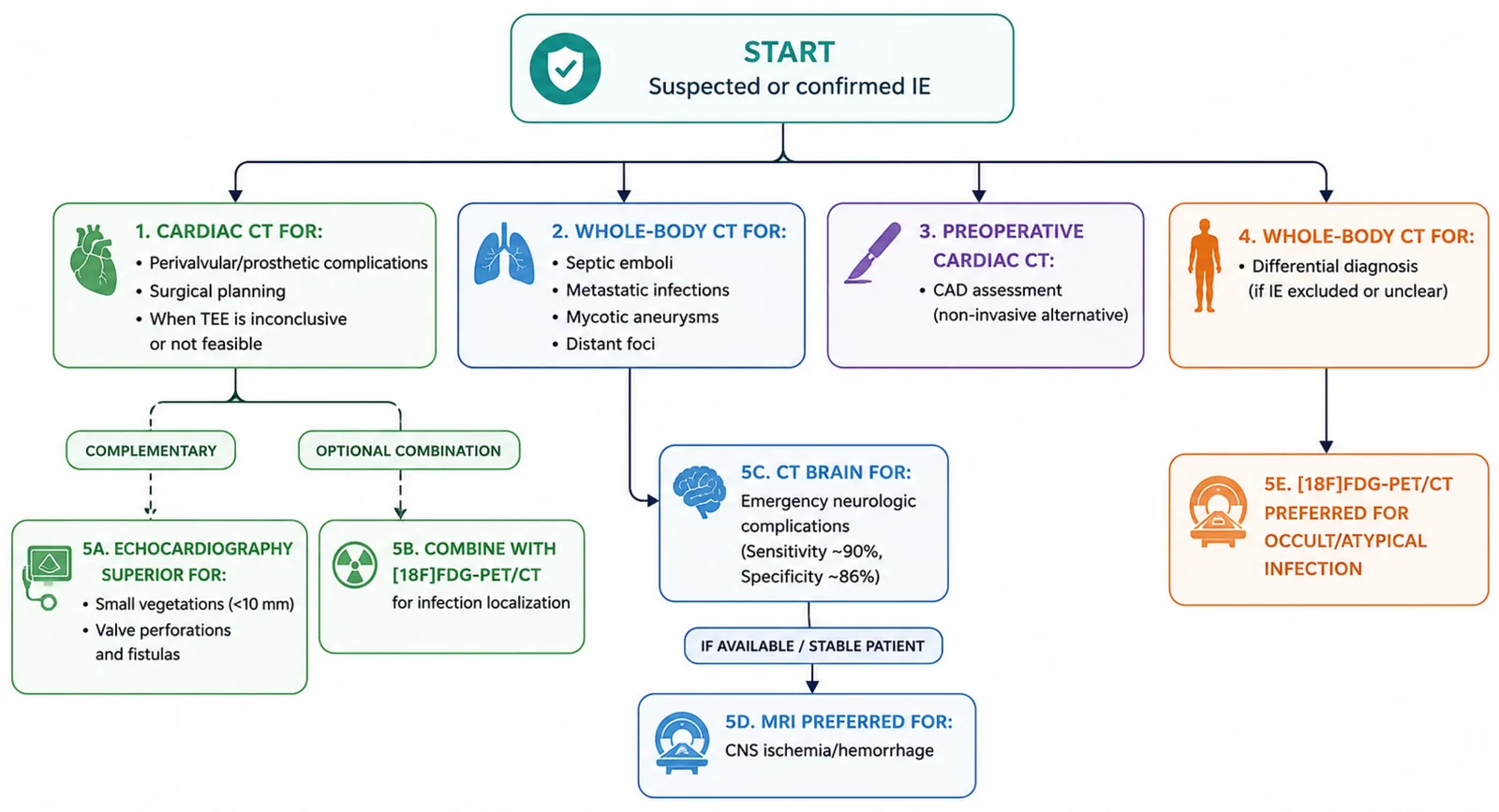
CT flow chart.

**Figure 4:**
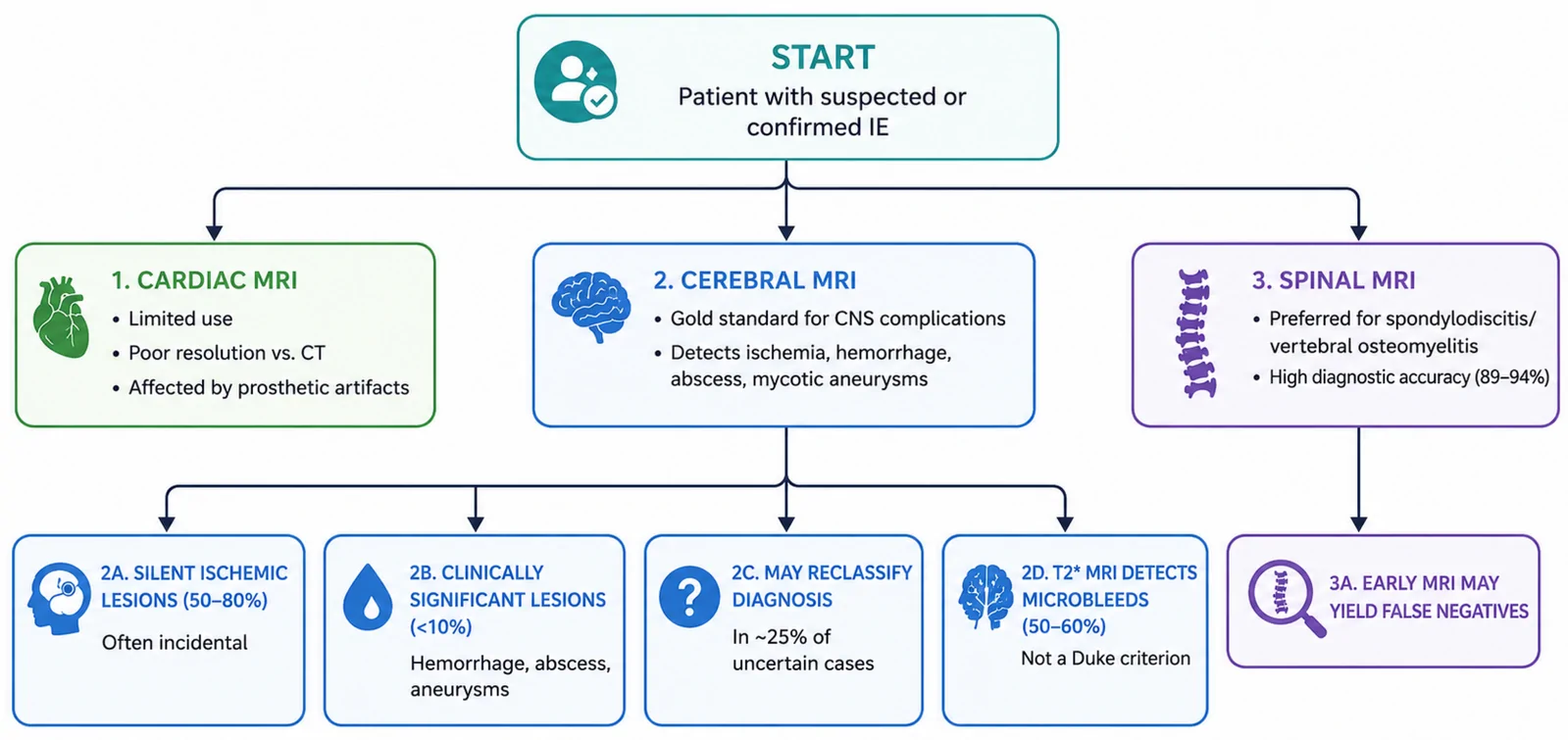
MRI flow chart.

Multimodality imaging plays a central role in the diagnosis, risk stratification, and management of infective endocarditis (IE). While echocardiography remains the first-line imaging modality, computed tomography (CT) and magnetic resonance imaging (MRI) provide complementary information, particularly for detecting extracardiac complications, evaluating surgical candidates, and improving diagnostic accuracy in selected patients. Echocardiography, including transthoracic (TTE) and transesophageal (TEE) imaging, remains the cornerstone of IE diagnosis. It enables the assessment of vegetations, valve morphology, leaflet perforation, intracardiac fistulas, and perivalvular complications such as abscesses, pseudoaneurysms, and prosthetic valve dehiscence. Vegetation size, particularly maximal length, is a key parameter for surgical decision-making. Although TTE is recommended as the initial examination, its sensitivity is limited, especially in prosthetic valves, small vegetations, device-related infections, and perivalvular extension. Therefore, TEE is recommended when TTE is negative or inconclusive despite high clinical suspicion, or when TTE confirms IE but further anatomical definition or complication assessment is required. Early imaging is essential because delayed diagnosis increases the risk of valvular destruction, embolic events, and surgery. In patients with persistent suspicion or high-risk features, repeat echocardiography within 5–7 days is recommended if the initial examination is nondiagnostic. During follow-up, repeat TTE or TEE may be useful to monitor vegetation evolution and detect new complications. Although several clinical scores have been proposed to identify bacteremic patients requiring echocardiography—particularly in Staphylococcus aureus bacteremia—their routine implementation remains under investigation [44,45,46,47,48,49,50,51,52,53,54,55,56,57,58] (Figure 2). Cardiac CT is an important complementary technique when echocardiography is inconclusive or technically limited. It is superior to TEE for detecting perivalvular and prosthetic complications, including abscesses, pseudoaneurysms, and fistulas, while also providing detailed anatomical information for surgical planning. However, echocardiography remains more sensitive for identifying small vegetations, leaflet perforations, and highly mobile lesions. Cardiac CT may be performed alone or combined with [^18^F]FDG-PET/CT, particularly in prosthetic valve endocarditis. Whole-body CT is valuable for detecting septic emboli, metastatic infections, mycotic aneurysms, and other distant infectious foci that may influence both Duke criteria classification and therapeutic strategy. In emergency settings, brain CT represents a practical alternative to MRI for identifying ischemic and hemorrhagic complications, although MRI remains the reference standard. In patients undergoing surgery, cardiac CT also provides a reliable non-invasive assessment of coronary artery disease, reducing the need for invasive coronary angiography in selected cases. When IE is excluded, or diagnostic uncertainty persists, whole-body CT may identify alternative infectious sources, whereas [^18^F]FDG-PET/CT is generally preferred for detecting occult or atypical infections [59,60,61,62,63,64,65,66] (Figure 3). MRI has a more selective role in IE. Cardiac MRI is not routinely recommended because of its lower spatial resolution compared with CT and susceptibility to prosthetic artifacts. In contrast, cerebral MRI is the gold standard for detecting neurological complications, including ischemic lesions, hemorrhage, cerebral abscesses, and mycotic aneurysms. Silent cerebral ischemic lesions are common, whereas clinically significant complications occur less frequently but may substantially influence therapeutic decisions. Routine brain MRI may also improve diagnostic classification by identifying embolic lesions fulfilling Duke minor criteria, reclassifying up to one-quarter of patients with initially uncertain diagnoses. T2*-weighted sequences frequently detect cerebral microbleeds, although these findings are not currently included among the Duke criteria. Finally, spinal MRI is the imaging modality of choice for diagnosing spondylodiscitis and vertebral osteomyelitis, with high diagnostic accuracy, although very early examinations may occasionally be falsely negative. Overall, the integration of echocardiography with advanced imaging modalities has significantly improved the diagnostic pathway of IE, allowing more accurate assessment of cardiac lesions, systemic complications, and surgical planning while supporting a personalized, multidisciplinary management strategy [67,68,69,70,71,72,73,74] (Figure 4).

#### Echocardiography vs. CT

The diagnosis of infective endocarditis (IE) is based on the modified Duke criteria, which incorporate clinical, microbiological, and echocardiographic findings. Transthoracic echocardiography (TTE) is the initial imaging modality of choice; however, it has limited sensitivity, particularly in early-stage disease or when vegetations are small. Consequently, transesophageal echocardiography (TEE) is currently regarded as the reference standard for the morphological and functional assessment of cardiac structures in patients with suspected IE [3]. Despite its widespread use and high diagnostic yield, TEE has inherent limitations. Its accuracy may be influenced by operator expertise, patient anatomy, and imaging artifacts caused by valvular calcifications or mechanical prostheses—factors that are particularly relevant in the evaluation of anterior aortic abscesses. Moreover, TEE may be contraindicated (e.g., in patients with esophageal disease) or poorly tolerated. In up to 30% of patients with confirmed IE, echocardiographic findings may be negative or inconclusive. In this setting, cardiac computed tomography (cardiac CT) has garnered increasing interest due to its high diagnostic accuracy in detecting valvular and perivalvular complications, including abscesses, pseudoaneurysms, leaflet perforations, and paravalvular leaks. Recent systematic reviews—albeit based on limited case series—have suggested that cardiac CT could be considered as part of a multimodal diagnostic approach to IE. Nevertheless, according to current international guidelines, cardiac CT is recommended only in selected cases in which TTE and TEE are inconclusive or technically infeasible. At present, no formal recommendations support its routine use once the diagnosis of IE has been established. Oliveira et al. [49] considered 8 studies that met the inclusion criteria. Comparative analysis demonstrated that cardiac computed tomography (CT) exhibited greater sensitivity than transesophageal echocardiography (TEE) in the detection of abscesses or pseudoaneurysms, with values of 78% (95% confidence interval [CI]: 70–85%; 112 of 142) versus 69% (95% CI: 62–76%; 94 of 135), respectively, a difference approaching statistical significance (*p* = 0.052). In studies employing multiphase CT protocols, sensitivity increased further to 87% (95% CI: 78–93%; 70 of 79), a difference that reached statistical significance when compared to TEE (*p* = 0.04). Conversely, TEE demonstrated significantly higher sensitivity than CT in the detection of endocardial vegetations, with rates of 94% (95% CI: 92–96%; 363 of 383) versus 64% (95% CI: 57–70%; 151 of 237), respectively (*p* < 0.001). A similar trend was observed for the detection of valvular leaflet perforations, with a sensitivity of 81% for TEE (95% CI: 71–88%; 74 of 91) compared to 41% for CT (95% CI: 25–59%; 14 of 35) (*p* = 0.02). With regard to the diagnosis of paravalvular leakage, sensitivity was 69% for TEE (95% CI: 58–79%; 56 of 80) and 44% for CT (95% CI: 30–59%; 21 of 48), although this difference did not reach statistical significance (*p* = 0.27). Cardiac CT demonstrated superior diagnostic performance compared to TEE for the detection of abscesses and pseudoaneurysms. In contrast, TEE remains more accurate for the identification of vegetations, leaflet perforations, and paravalvular leaks (Table 1).

**Figure 5:**
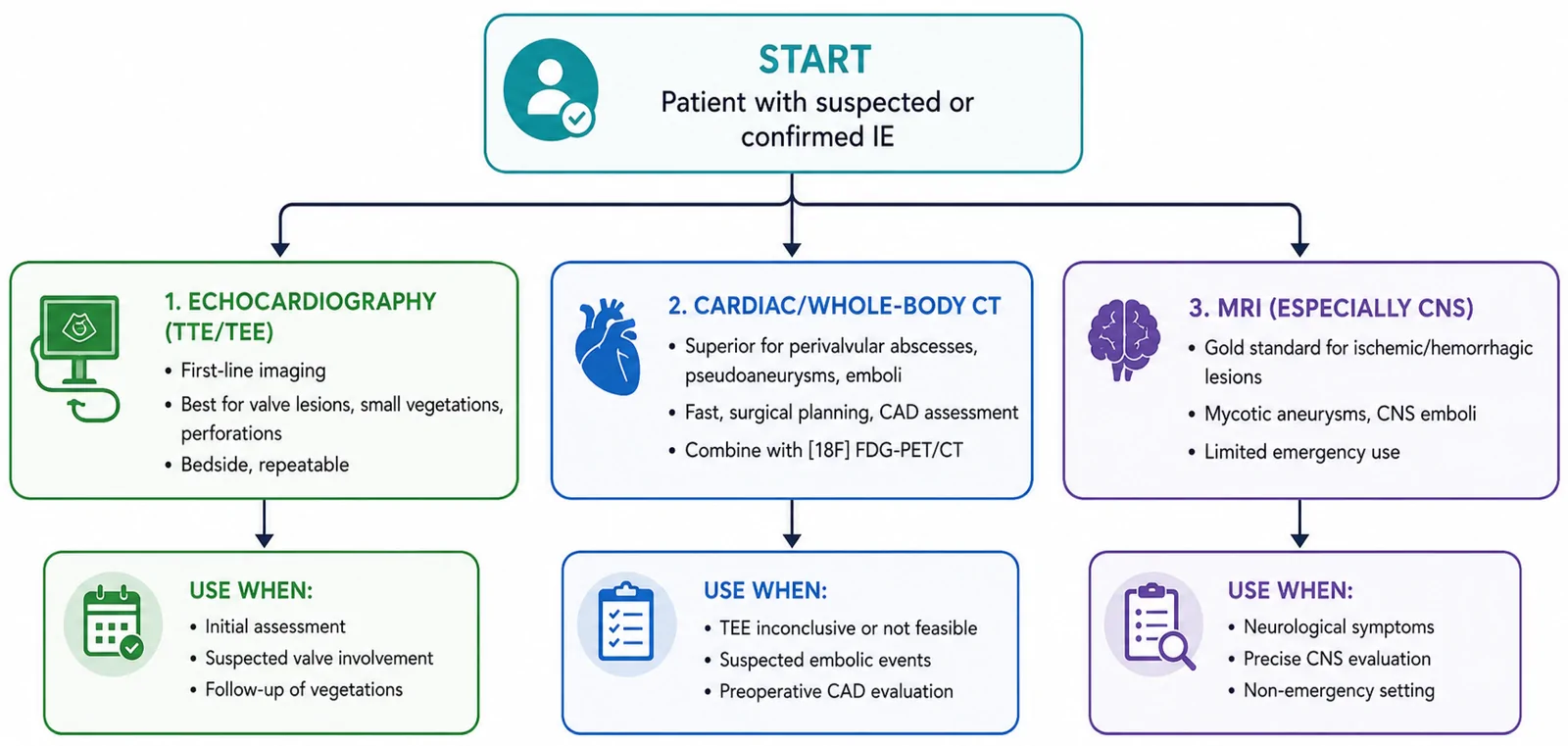
Comparison between Echo, CT and MRI.

Jain et al. [48] analyzed data that show that the pooled sensitivity and specificity of transesophageal echocardiography (TEE) for vegetation detection were 96% and 83%, respectively, whereas cardiac computed tomography (CT) yielded values of 85% and 84%. In the subgroup of patients with prosthetic valves, the pooled sensitivity and specificity of TEE for vegetation detection were 89% and 74%, respectively, compared to 78% and 94% for CT, with CT demonstrating significantly higher specificity (*p* < 0.05). Regarding perianular complications, the pooled sensitivity and specificity of TEE were 70% and 96%, respectively, while CT showed higher sensitivity (88%) and comparable specificity (93%). Although the difference in sensitivity did not reach statistical significance (*p* = 0.06), CT demonstrated a trend toward improved performance in detecting perianular involvement. In the detection of leaflet perforations, TEE showed a sensitivity of 79% and a specificity of 93%, compared to 48% sensitivity and 93% specificity for CT. The superiority of TEE in this context was statistically significant (*p* < 0.05). For the diagnosis of fistulas, paravalvular regurgitation, and prosthetic valve dehiscence, the two imaging modalities showed comparable diagnostic performance. Overall, both TEE and CT demonstrated good diagnostic accuracy for valvular involvement and complications related to infective endocarditis. TEE proved to be more effective in identifying leaflet abnormalities, whereas CT performed better in the evaluation of prosthetic valve involvement and showed a trend toward greater sensitivity in detecting perianular complications. The appropriate and complementary use of TEE and CT, within a multimodality imaging strategy, may enhance diagnostic performance in clinical practice (Table 2, Figure 5).

## 4. Indications for Surgery

Surgery is required in a substantial proportion of patients with infective endocarditis (IE) and provides a significant survival benefit compared with medical therapy alone. During active IE, surgical indications are primarily based on three clinical scenarios: heart failure (HF), uncontrolled infection, and prevention of embolic events. Depending on clinical severity, surgery may be performed on an emergency basis (within 24 h), urgently (within 3–5 days), or electively during hospitalization. After infection resolution, surgical indications should follow standard valvular heart disease guidelines. Although surgery during active infection carries increased operative risk, age or comorbidities alone should not preclude intervention. Decisions regarding surgical futility require individualized multidisciplinary assessment, taking into account patient preferences and overall prognosis. Heart failure is the most common complication and the leading indication for urgent surgery. It usually results from severe valvular dysfunction caused by leaflet or chordal rupture, large vegetations, abscesses, or intracardiac fistulas, and may range from mild symptoms to cardiogenic shock. Echocardiography, supported by cardiac biomarkers, is essential for hemodynamic assessment. Emergency surgery is indicated in patients with pulmonary edema or cardiogenic shock, whereas urgent intervention is recommended for severe valve regurgitation associated with heart failure or echocardiographic evidence of hemodynamic compromise. Uncontrolled infection is characterized by persistent fever or positive blood cultures despite appropriate antimicrobial therapy, particularly when accompanied by increasing vegetation size, abscess formation, pseudoaneurysms, fistulas, prosthetic valve involvement, or new conduction abnormalities. Perivalvular extension occurs more frequently in aortic and prosthetic valve endocarditis. Transesophageal echocardiography remains the reference imaging modality, while cardiac CT and [^18^F]FDG-PET/CT provide complementary information, particularly in prosthetic valve infections.

Prevention of embolic events represents the third major indication for surgery. Embolic complications most commonly involve the brain, spleen, and lungs, with the highest risk occurring before and during the first days of antibiotic therapy. Large, mobile mitral vegetations, previous embolic events, multivalvular infection, and highly virulent pathogens identify patients at greatest embolic risk, in whom early surgery (within 3–5 days) should be strongly considered. Overall, optimal surgical management of IE requires a multidisciplinary Heart Team approach integrating clinical, microbiological, echocardiographic, and multimodality imaging findings with individualized operative risk assessment [75,76,77,78,79,80,81,82,83,84].

### IE with Neurological Damage

Neurological complications are among the most severe manifestations of infective endocarditis (IE). Embolic events occur in up to 50% of patients, with cerebral embolism causing stroke in approximately 15–30% of cases and significantly increasing morbidity and mortality. Clinical presentation varies according to the type and extent of cerebral injury, which may be ischemic, hemorrhagic, or clinically silent. When cardiac surgery is indicated, it should not be delayed after minor neurological events, such as silent emboli or transient ischemic attacks. In contrast, severe neurological injury or intracranial hemorrhage generally warrants postponement of surgery. Current practice recommends delaying surgery for approximately four weeks after intracerebral hemorrhage, although shorter intervals (≥1 week) may be considered in carefully selected patients, balancing neurological risk against the need for urgent infection control [85,86,87,88,89,90,91,92,93,94,95]. Although several studies have reported acceptable outcomes with early cardiac surgery performed within days of hemorrhagic stroke, the optimal timing of intervention in patients with infective endocarditis (IE) remains controversial and is not supported by standardized recommendations. Surgical timing should therefore be individualized through a multidisciplinary Heart Team approach, balancing neurological risk against the need for infection control and cardiac intervention [6,96,97,98,99,100,101,102,103,104,105,106]. Dashkevich et al. [107] analyzed 119 patients with infective endocarditis (IE) complicated by cerebral embolism, stratified according to surgical timing: early (1–7 days), intermediate (8–21 days), and late (>22 days). Overall in-hospital mortality was 15.1%, with the highest rate observed in the intermediate group (25.7%). Univariate analysis identified advanced age, preoperative respiratory and renal failure, large vegetations, and delayed surgery as predictors of in-hospital mortality. However, multivariate analysis showed that vegetation size >8 mm and advanced age were the only independent predictors, whereas surgical timing was not. These findings suggest that early surgery should be considered in patients with large vegetations, irrespective of neurological injury severity. Although postponing surgery may be appropriate in selected patients to allow neurological stabilization, excessive delays may result in disease progression, worsening heart failure, and increased in-hospital mortality.

## 5. Surgical Scope

### 5.1. Valve Function Restoration

**Figure 6:**
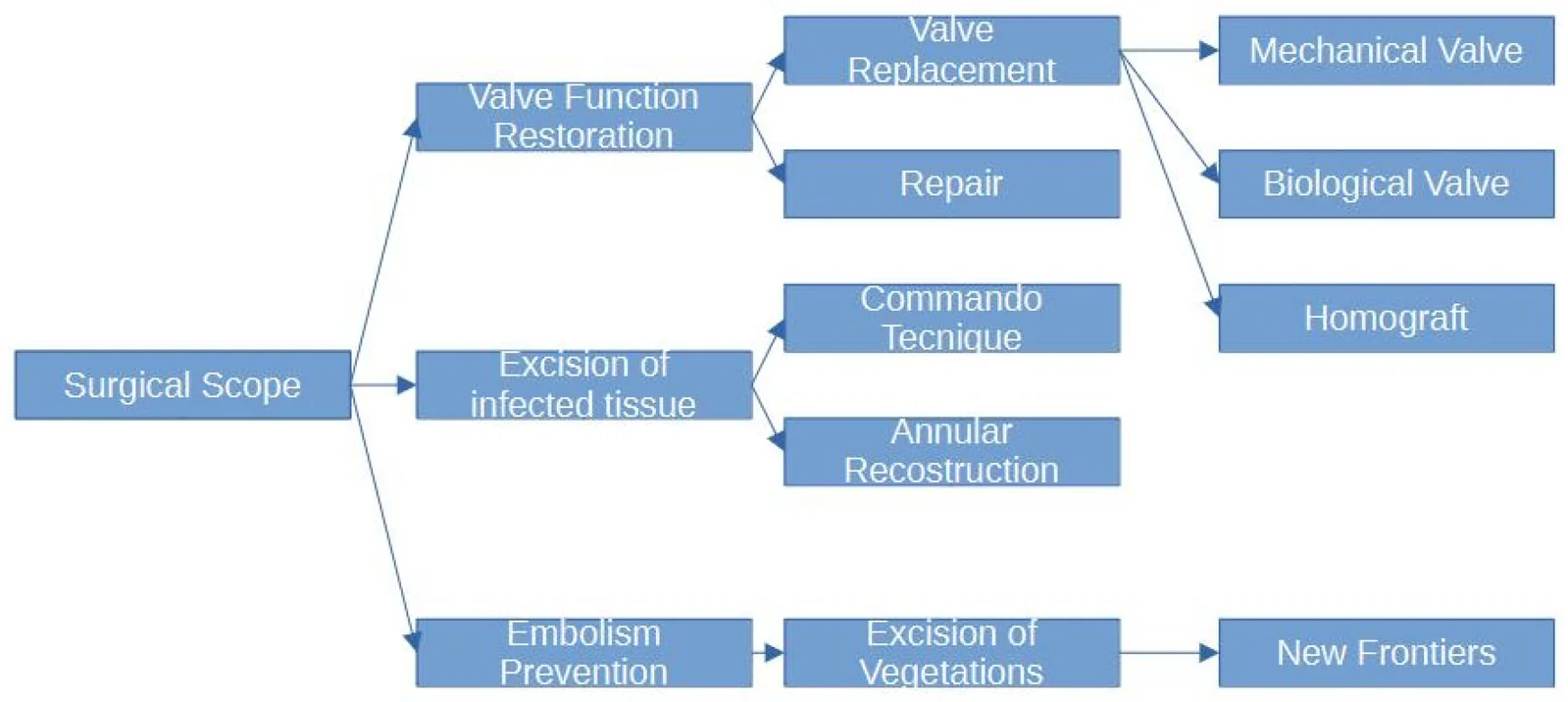
Ideal Surgical scope.

**Figure 7:**
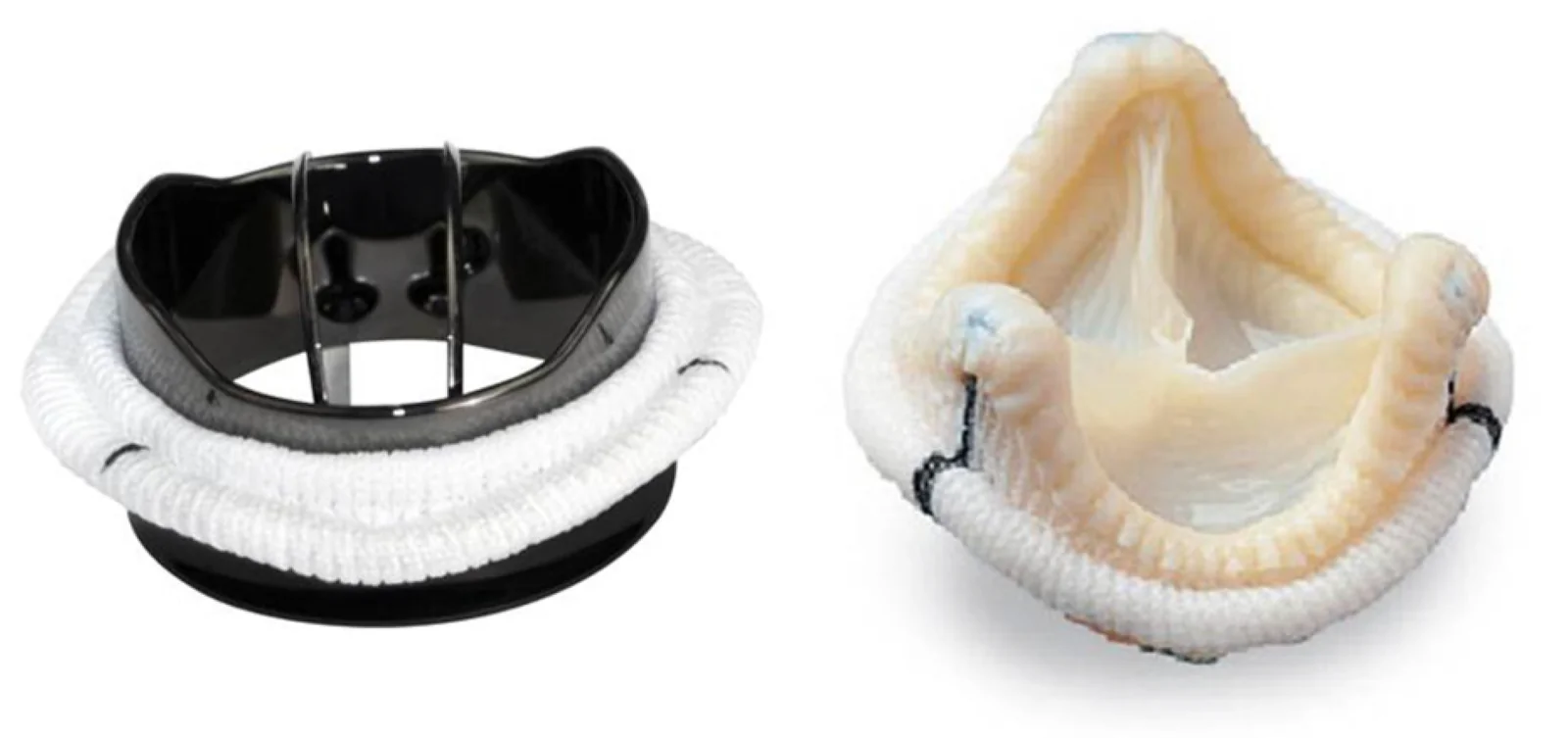
Mechanical valve and Biological valve.

Since the first successful aortic valve replacement (AVR) with a mechanical prosthesis in the 1960s, prosthetic valve technology has evolved considerably. Early caged-ball valves have been replaced by tilting-disc and bileaflet mechanical prostheses, which provide superior hemodynamic performance and lower thrombogenicity. Nevertheless, all mechanical valves require lifelong anticoagulation with vitamin K antagonists (VKAs) to prevent thromboembolic complications related to Virchow’s triad, at the expense of an increased bleeding risk. Owing to their excellent durability, often exceeding 20 years, mechanical valves remain the preferred option for patients younger than 50 years without contraindications to anticoagulation. Bioprosthetic valves, derived mainly from bovine pericardium or porcine tissue, are available in stented and stentless designs. Their principal limitation is structural valve degeneration (SVD), primarily driven by progressive tissue calcification, which reduces valve durability. Consequently, bioprostheses are generally recommended for patients older than 65 years or those unsuitable for long-term anticoagulation. As the use of biological valves continues to increase, improving the understanding of the mechanisms underlying SVD has become essential for enhancing prosthetic durability and long-term clinical outcomes (Figure 6 and Figure 7).

Another fundamental technique is homograft implantation. As early as 2007, Musci et al. [108] described the effectiveness of this approach. This study reports a 20-year, single-center experience involving a cohort of high-risk patients diagnosed with active infective endocarditis, affecting either native (NVE) or prosthetic (PVE) valves, who underwent aortic root replacement (ARR) with cryopreserved homografts. A comparative analysis was performed to evaluate clinical outcomes between these two patient populations. The results indicate that patients with PVE exhibited significantly poorer outcomes compared to those with NVE, as reflected by higher operative mortality and decreased long-term survival. Analysis of the survival curves revealed a particularly pronounced difference within the first 30 postoperative days. It may be inferred that the more favorable outcomes observed in the NVE group are, at least in part, attributable to the more advanced stage of infection and the more critical clinical condition typically observed in patients with PVE at the time of surgery. Patients in the PVE cohort were not only significantly older, but also had a higher prevalence of preoperative renal insufficiency and infections caused by Staphylococcus species. In addition, periannular abscess formation with severe destruction of the aortic root was more frequently encountered in this group, resulting in a substantially higher incidence of aorto-ventricular dehiscence compared to NVE patients (69.7% vs. 35.4%). Each of these factors contributes to the increased clinical complexity and heterogeneity associated with active infective endocarditis involving prosthetic valves and may, collectively, explain the elevated morbidity and mortality observed in this subgroup. Despite the high operative morbidity and mortality associated with these procedures, long-term survival outcomes were encouraging, especially when considering the critical preoperative condition of the patients and the technical complexity of the interventions performed. The low incidence of early reinfection and the excellent long-term freedom from reinfection—observed consistently across both native valve endocarditis (NVE) and prosthetic valve endocarditis (PVE) groups—support the pivotal role of cryopreserved homografts in the management and eradication of active infective endocarditis (AIE). In cases of extensive tissue destruction with associated aorto-ventricular dehiscence, anatomical reconstruction can be effectively achieved by incorporating the muscular cuff of the annulus and the anterior mitral leaflet into the homograft reconstruction, thereby restoring structural integrity. The observed reduction in infection-related risk may, in part, be attributed to the use of homografts, which, due to their natural biocompatibility, demonstrate a greater resistance to reinfection compared to other prosthetic materials. When interpreting outcomes from homograft-based surgical series, it is essential to clearly report the incidence of active infective endocarditis and associated abscesses within the study population, as well as to detail the specific surgical techniques employed. This surgical approach allows for thorough infection control through radical debridement and complete replacement of all infected tissue within the aortic annulus and root. Additionally, it facilitates reconstruction of the left ventricular outflow tract, which is particularly beneficial in advanced cases of destructive endocarditis complicated by aorto-ventricular dehiscence. Homograft aortic root replacement (ARR) has been consistently associated with a low incidence of recurrent endocarditis. However, the long term durability of the homograft may be compromised by structural valve deterioration (SVD), a risk that becomes more pronounced in younger patients. Consequently, reoperation in these cases remains a significant clinical challenge. Despite being introduced into clinical practice over 45 years ago, homograft ARR continues to be the preferred valve substitute at our institution for the treatment of active infective endocarditis with periannular abscess formation [109].

In a study published in 2015, Kim et al. [110] conducted a comprehensive analysis of prospectively collected data from two tertiary academic centers, identifying a cohort of 304 consecutive adult patients (aged ≥ 17 years) who underwent surgical treatment for active infective endocarditis (IE) involving the aortic valve between 2002 and 2014. Short- and long-term clinical outcomes were evaluated using propensity score matching and inverse probability of treatment weighting to minimize selection bias and ensure robust comparative analysis. Valve replacement was performed using cryopreserved homografts in 86 patients (28.3%), xenograft bioprostheses in 139 patients (45.7%), and mechanical prostheses in 79 patients (26.0%). Homografts were more commonly employed in the context of prosthetic valve endocarditis (58.1% vs. 28.8%, *p* = 0.002) and in cases involving methicillin-resistant Staphylococcus species (25.6% vs. 12.1%, *p* = 0.002), when compared to conventional prosthetic options. Early postoperative mortality was significantly higher in the homograft group (19.8%) relative to the conventional prosthesis group (9.2%; *p* = 0.019). During the follow-up period (median: 29.4 months; interquartile range: 4.7–72.6 months), 60 patients (19.7%) died and 23 (7.7%) developed recurrent infection. However, no statistically significant differences were observed in overall survival (*p* = 0.23) or freedom from reinfection (*p* = 0.65) based on the type of prosthesis implanted. Following adjustment for baseline clinical variables through propensity score analysis, the use of homografts did not significantly impact early mortality (odds ratio [OR] 1.61; 95% confidence interval [CI]: 0.73–3.40; *p* = 0.23), overall mortality (hazard ratio [HR] 1.10; 95% CI: 0.62–1.94; *p* = 0.75), or risk of reinfection (HR 1.04; 95% CI: 0.49–2.18; *p* = 0.93). Did not demonstrate a statistically significant advantage of homograft use in reducing the risk of reinfection in patients undergoing surgery for active infective endocarditis. The selection of the most appropriate prosthetic valve should therefore be guided by technical considerations and patient-specific factors. Notably, the unavailability of homografts should not be regarded as a contraindication to timely and appropriate surgical intervention (Table 3).

### 5.2. Excision of Infected Tissue

The concomitant replacement of the aortic and mitral valves, combined with reconstruction of the aortomitral fibrous continuity, represents one of the most technically demanding procedures in contemporary cardiac surgery. Commonly referred to as the “Commando” or “UFO” procedure, this operation is indicated in cases of extensive structural disruption involving the intervalvular fibrous body—a critical anatomical component of the heart’s central fibrous skeleton.

This region, located between the medial and lateral fibrous trigones, plays a fundamental role in maintaining the structural integrity of the left heart and is situated at the junction of the anterior mitral leaflet, the left and non-coronary aortic sinuses, and the roof of the left atrium. Pathologies affecting this area often compromise both valvular structures, necessitating complex surgical intervention.

The most frequent indication for reconstruction of the aortomitral curtain is advanced infective endocarditis, characterized by extensive tissue destruction, annular abscess formation, and involvement of both the aortic and mitral valves. Additional indications may include severe anterior mitral annular calcification, the need for annular enlargement in the setting of double-valve replacement, and structural degeneration resulting from multiple previous valve surgeries.

The surgical strategy described herein reflects the standardized technique currently adopted by the majority of cardiac surgeons at our institution for combined aortic and mitral valve replacement with comprehensive reconstruction of the aortomitral fibrous continuity [111,112,113,114,115,116,117].

Following initiation of cardiopulmonary bypass and myocardial arrest—achieved via antegrade administration of del Nido cardioplegia—the ascending aorta is incised in an oblique trajectory, extending into the midpoint of the non-coronary sinus to enhance visualization and access. Upon aortotomy, the aortic valve is thoroughly inspected, and radical debridement of all infected and devitalized tissue is performed.

In patients with a previously implanted prosthetic or transcatheter aortic valve, explantation is meticulously carried out by circumferential dissection along the native annular plane. The aortotomy is then extended across the aortic annulus onto the dome of the left atrium and the central segment of the anterior mitral leaflet. A longitudinal atriotomy is directed toward the right superior pulmonary vein to expose the atrial chamber and facilitate complete resection of pathological tissue.

In the presence of structural compromise involving the aortomitral curtain, en bloc excision of the entire intervalvular fibrous body—extending from the left to the right fibrous trigone—is undertaken to ensure complete eradication of the infectious process. The mitral valve is assessed, with resection of the anterior leaflet and associated subvalvular apparatus routinely performed. Preservation of the posterior leaflet and chordae is prioritized when anatomically feasible, in order to maintain left ventricular geometry and preserve ventricular function. If a mitral prosthesis is present, it is explanted and the annulus is subjected to extensive debridement.

Reconstruction begins with mitral valve replacement. Valve sizing is performed to ensure that the anterior aspect of the sewing ring lies inferior to the plane of the aortic annulus, thereby facilitating subsequent aortic prosthesis seating. Oversizing is strictly avoided to mitigate the risk of left ventricular outflow tract obstruction. Interrupted pledgeted sutures are placed along the posterior annulus from trigone to trigone, and the mitral prosthesis is parachuted into position and secured.

Subsequently, a bovine pericardial patch—typically tailored in a surfboard configuration—is employed to reconstruct the aortomitral curtain, the superior dome of the left atrium, and, if necessary, the aortic root. The patch is initially sized according to the longitudinal intertrigonal distance and later adjusted to the patient’s specific anatomy. A width approximately 1 cm greater than the measured trigone-to-trigone distance is selected to provide sufficient tissue for anchoring and contouring around the anterior aspect of the mitral prosthesis.

The patch is affixed to the anterior mitral sewing ring using interrupted or continuous polypropylene sutures. Particular attention is paid to achieving precise anatomical alignment among the fibrous trigones, the prosthetic valve, and the pericardial patch to minimize the risk of perivalvular bleeding. Reinforcement at the trigonal junctions is routinely performed using figure-of-eight sutures to prevent potentially fatal hemorrhagic complications. The inferior segment of the patch is incorporated into the left atrial roof to complete the closure of the atriotomy with bilateral running sutures.

The necessity for aortic root replacement is determined intraoperatively based on the extent of infection and the structural integrity of the coronary sinuses. If indicated, coronary buttons are mobilized, and a valved conduit is implanted following conventional Bentall principles. Alternatively, in cases of severely compromised annular tissue, an aortic homograft may be utilized.

In patients undergoing root replacement, the superior portion of the pericardial patch is trimmed to accommodate annular suture placement along the reconstructed non-coronary sinus. When root replacement is not required, the pericardial patch is directly incorporated into the native aortic annulus using interrupted sutures to recreate the non-coronary sinus. Care is taken to avoid excessive spatial separation between the mitral and aortic suture lines along the patch to prevent asynchronous movement or “rocking” of the prosthetic valves.

**Figure 8:**
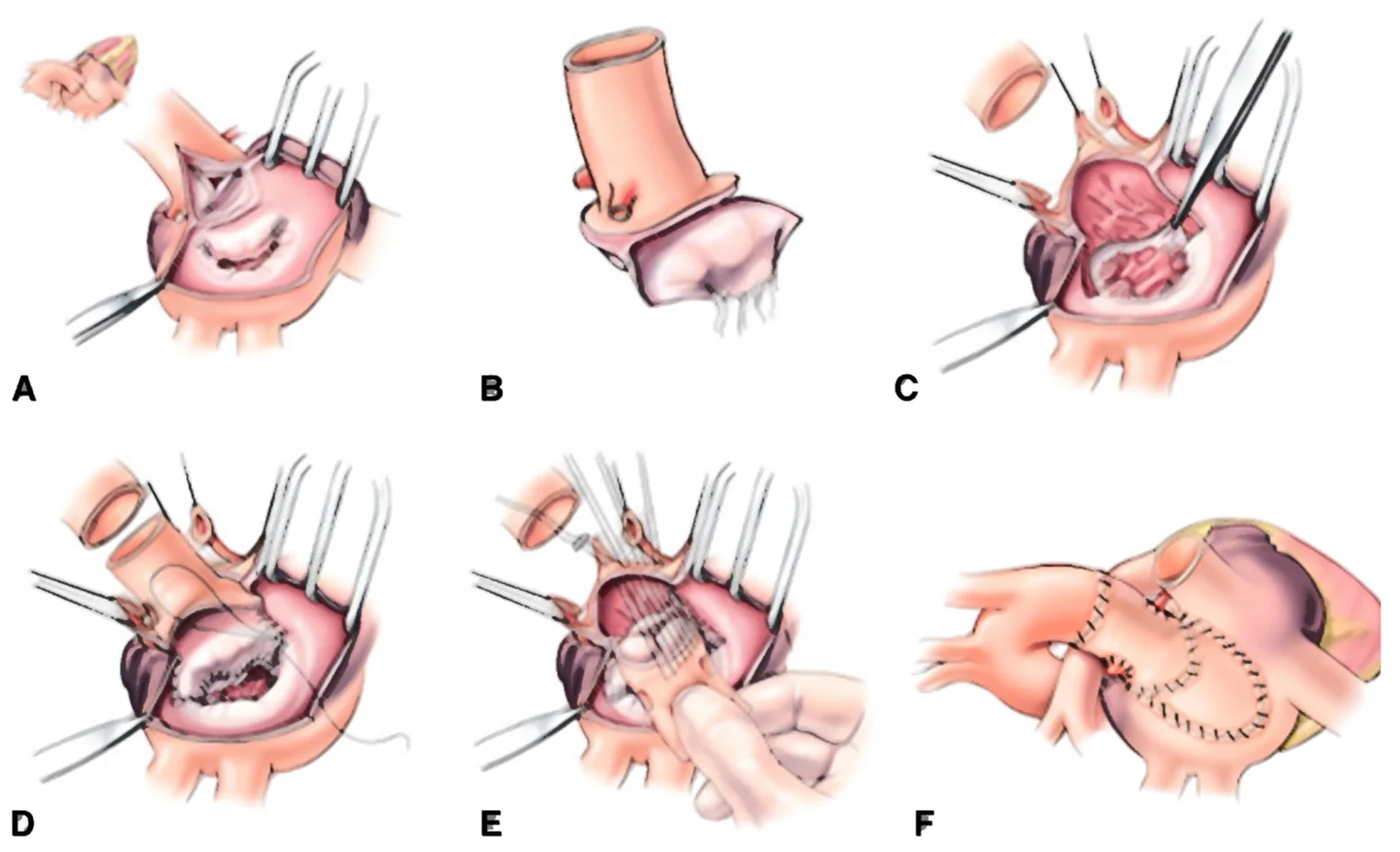
(**A**–**F**) Commando procedure.

Final reconstruction of the non-coronary sinus is completed using the superior half of the pericardial patch, and the aortotomy is closed with a continuous polypropylene suture in standard fashion [118] (Figure 8).

In a retrospective multicenter analysis, Forteza-Gil at al. [117] examined the outcomes of patients who underwent intervalvular fibrous body reconstruction due to acute infective endocarditis, treated at two national referral centers between April 2014 and November 2023. Participants were stratified into two groups according to the extent of aortic root involvement: those undergoing conventional intervalvular reconstruction without aortic root replacement (non-ROOT or “Commando” group), and those in whom the procedure included aortic root replacement (“Root-Commando” or ROOT group). A total of 78 patients met the inclusion criteria—30 (38.5%) in the ROOT group and 48 (61.5%) in the non-ROOT group. Comparative analysis revealed no statistically significant differences between the two cohorts in terms of perioperative mortality, incidence of postoperative complications, reinfection rates, or the need for subsequent surgical reinterventions. Notably, no cases of relapse were documented in either group. The median follow-up duration was 4.69 years (95% confidence interval: 3.10–5.13 years). The combined in-hospital and 30-day mortality rate was 17.9% (14 patients), with no significant intergroup variation. Overall survival at 1 and 5 years was 76.2% and 67.2%, respectively. At the 1-, 2-, and 5-year marks, survival rates were 74%, 74%, and 68% in the non-ROOT cohort, and 79%, 79%, and 63% in the ROOT cohort, respectively. These findings reinforce the viability of Commando-type procedures, including those requiring concomitant aortic root replacement, as a means of achieving durable structural reconstruction in high-risk patients for whom standard reparative options are not feasible. The data suggest that, when surgical reconstruction is successfully accomplished, the choice to replace the aortic root does not negatively impact short- to mid-term survival outcomes. Moreover, the absence of recurrent infections supports the efficacy of these complex surgical approaches in eradicating active infective endocarditis (Table 4).

### 5.3. Embolism Prevention—New Frontiers

The prevention of septic embolism remains a key component in the therapeutic management of valvular infective endocarditis. While conventional surgery continues to represent the gold standard of treatment, novel and progressively less invasive techniques have emerged in recent years. However, these approaches are not yet fully standardized nor widely adopted in clinical practice. A particularly challenging clinical scenario involves the presence of vegetations on the tricuspid valve. Tricuspid valve endocarditis is frequently observed in patients with a history of intravenous drug use—a population characterized by a high rate of recurrent infections and poor adherence to long-term follow-up. In this specific patient subgroup, emerging therapeutic strategies that avoid the use of prosthetic materials—such as valve-sparing approaches or temporary biological valves—may offer viable treatment alternatives. These non-prosthetic strategies could help reduce the risk of reinfection and may be more suitable for high-risk cohorts. Although tricuspid valve disease is not the primary focus of this review, it presents a relevant clinical consideration. It is conceivable that percutaneous approaches currently used for tricuspid endocarditis could, in the future, be extended to patients with small vegetations (<10 mm) on the aortic valve, particularly in cases not indicated for surgery or unresponsive to antibiotic therapy. Accordingly, we believe it is of interest to critically examine the current literature regarding percutaneous management of infective endocarditis involving the tricuspid valve.

Suruagy-Motta et al. [118] conducted a systematic literature review aimed at comparing the clinical outcomes of percutaneous mechanical aspiration (PMA) versus conventional surgery in the treatment of tricuspid valve infective endocarditis (TVIE). The search spanned five major bibliographic databases and included comparative studies evaluating both therapeutic strategies. The outcomes of interest comprised all-cause mortality at 30 days and one year, in-hospital mortality, length of hospital stay, and readmission rates due to recurrent endocarditis. Ten retrospective studies were included, encompassing a total of 6035 patients: 974 treated with PMA and 5061 undergoing surgical intervention. The analysis showed no significant difference in in-hospital mortality between the two groups (RR = 1.07; *p* = 0.91). However, patients treated with PMA had a significantly higher 30-day mortality rate (RR = 2.71; 95% CI: 1.53–4.82; *p* < 0.001). No statistically significant differences were observed for one-year mortality (RR = 1.13; 95% CI: 0.72–1.77; *p* = 0.60) or for readmissions due to endocarditis (RR = 0.82; *p* = 0.63). Notably, PMA was associated with a significantly shorter hospital stay compared to surgery (MD = −7.0 days; 95% CI: −13.0 to −1.1; *p* = 0.03). These findings suggest that while PMA may offer a shorter hospitalization period, conventional surgical management ensures better short-term survival in patients with TVIE. Given the comparable long-term outcomes between the two modalities, well-designed prospective randomized trials are warranted to further define the risk–benefit profile of these therapeutic options in this high-risk population (Table 5).

A retrospective cohort analysis conducted by Veve et al. [119] examined adult individuals with a history of intravenous drug use (PWID) who were hospitalized with a definitive diagnosis of tricuspid valve infective endocarditis (TVIE) and subsequently treated with either percutaneous mechanical aspiration (PMA) or surgical valve replacement between January 2014 and April 2019. The study included a total of 85 patients: 42 underwent PMA, while 43 received conventional surgical management. Baseline demographic and clinical characteristics were comparable between the two cohorts. The majority of patients were female (*n* = 62, 73%), with a median age of 31 years (interquartile range: 27–41). A previous history of infective endocarditis was documented in 74 patients (86%), all of whom had received prolonged antibiotic therapy prior to intervention. Notably, 33 patients (38%) presented with septic shock at the time of admission. Methicillin-resistant Staphylococcus aureus (MRSA) was the most frequently isolated pathogen, identified in 38% of cases (32 out of 84). In-hospital mortality was observed in 5 patients (12%) treated with PMA and in 1 patient (2%) who underwent surgery (*p* = 0.11). Twelve-month all-cause mortality was 24% in the PMA group and 19% in the surgical group (*p* = 0.57). After adjustment for potential confounding variables, no statistically significant difference was found in 12-month mortality between the two treatment strategies (adjusted odds ratio = 1.5; 95% CI: 0.48–4.8). Likewise, secondary endpoints—including in-hospital mortality and readmission—did not differ significantly between the groups. These findings suggest that while surgical treatment may demonstrate a numerical advantage in short-term outcomes, percutaneous aspiration remains a viable therapeutic option in selected high-risk patients. Further prospective studies are warranted to delineate optimal treatment strategies for this complex patient population (Table 6).

## 6. Conclusions

Infective endocarditis remains one of the most significant challenges in contemporary medicine due to its aggressive course and the critical importance of timely intervention. A wide array of therapeutic options is currently available, and thanks to advances in pharmacology, antibiotics continue to serve as a highly effective weapon against this condition. Surgery, of course, remains a cornerstone in the management of endocarditis, with various techniques now widely validated and effectively employed. This review has focused on the most established and widely adopted surgical strategies. However, considering the heterogeneous and often unpredictable nature of infective endocarditis, we believe that any technique may be appropriate—provided it is applied to the right patient, at the right time. We also strongly support the formation of dedicated Endocarditis Teams, bringing together the expertise of multiple specialties—infectious diseases, cardiology, cardiac surgery, internal medicine, among others. Given the complexity and variability of the disease, a multidisciplinary approach is essential to achieve optimal clinical outcomes. While writing this article, a crucial reflection emerged: years of clinical experience and the review of numerous publications have shown that patients who have developed endocarditis—whether on a native or prosthetic valve—remain at ongoing risk for recurrence of the infection. This observation leads us to question whether the current direction of research—focused largely on improving therapies, surgical techniques, percutaneous approaches, and antibiotic protocols—is truly sufficient. While advancements in treatment and early diagnosis are undoubtedly essential, we believe it is time to shift focus toward prevention. In our opinion, the approach to patients at high risk of recurrent endocarditis needs to be re-evaluated. At present, a patient who has recovered from infective endocarditis—whether through antibiotic therapy or surgery—is often considered “cured.” We argue that this is a misleading perspective. Although the acute phase of the disease may be resolved, the patient remains at permanent risk of reinfection. For this reason, we propose that patients who have experienced endocarditis should be enrolled in a structured, long-term follow-up program, similar to the approach used for chronic diseases. To take this further, we put forward a provocative comparison: the management of patients who have had infective endocarditis should resemble that of oncology patients. Just as cancer patients undergo treatment followed by lifelong surveillance to detect treatable recurrences early, patients considered “cured” of infective endocarditis should also be closely monitored throughout their lives to prevent severe, destructive episodes that may carry unacceptably high surgical risk. We recognize that implementing follow-up protocols for at-risk patients is logistically challenging and resource-intensive, requiring significant investments in both healthcare personnel and infrastructure. Nonetheless, we firmly believe that such an approach could represent a critical advancement in the prevention of endocarditis, especially in high-risk populations.

## Figures and Tables

**Table 1:** TEE vs. CT.

Complication	TEE Sensitivity	CT Sensitivity	*p*-Value
Abscess/Pseudoaneurysm	69% (94/135)	78% (112/142) 87% (70/79)	0.052/0.04
Vegetation	94% (363/383)	64% (151/237)	<0.001
Leaflet Perforation	81% (74/91)	41% (14/35)	0.02
Paravalvular Leak	69% (56/80)	44% (21/48)	0.27

**Table 2:** TEE vs. Ct 2.

Findings	TEE Sensitivity/Specificity	CT Sensitivity/Specificity	Statistical Significance
Vegetation (overall)	96%/83%	85%/84%	TEE more sensitive
Vegetation (prosthetic valves)	89%/74%	78%/94%	CT more specific (*p* < 0.05)
Perianular complications	70%/96%	88%/93%	CT trend toward higher sensitivity (*p* = 0.06)
Leaflet perforation	79%/93%	48%/93%	TEE more sensitive (*p* < 0.05)
Paravalvular leak/Fistula/Dehiscence	Comparable	Comparable	No significant difference

**Table 3:** Comparison between the two studies.

Study Period	1986–2007	2002–2014
Total Patients	1163 total (221 with homograft)	304
Study type	Single-center, long-term	Two-center, prospective
Patient age	median 55 years	>17 years
Valve involved	Aortic root	Aortic valve
Homograft Use	100%	28.3%
Homograft use in PVE	122/221 (55%)	58.1% vs. 28.8% (*p* = 0.002)
Periannular Abscess	189/221 (86%)	Not specified
Aorto-ventricular Dehiscence	120/189 (63.5%)	Not specified
30-day Survival	83.8% ± 3.7%	Not reported
10-year Survival	47.3% ± 5.6%	Not reported
Reinfection Rate	5.4%	7.7%
Early Mortality	19.8%	19.8%
Reoperation Rate	14%	Not specified
Conclusion	Good long-term results	No significant benefit

**Table 4:** Data comparison.

Variable	ROOT Group	Non-ROOT Group	Overall
Total patiens	30 (38.5%)	48 (61.5%)	78
ROOT group (Commando + root replacement)	X		
non-ROOT group (Commando)		X	
Perioperative mortality	No significant difference	No significant difference	No significant difference
Postoperative complications	No significant difference	No significant difference	No significant difference
Reinterventions during follow-up	No significant difference	No significant difference	No significant difference
Reinfections during follow-up	No significant difference	No significant difference	No significant difference
Relapses	0	0	0
Median follow-up (years)	4.69 (95%)	4.69 (95%)	4.69 (95%)
in-hospital/30-day mortality	included in total	included in total	14 patiens (17.9%)
1-years survival	79%	74%	76.2%
2-years survival	79%	74%	not reported
5-years survival	63%	68%	67.2%

**Table 5:** Data comparison.

Outcome	PMA Group (*n* = 974)	Surgery Group (*n* = 5061)
in-hospital mortality	Similar to surgery (*p* = 0.91)	Similar to PMA
30-day mortality	Higher (*p* < 0.001)	Lower 30-day mortality
1-year mortality	No significant difference (*p* = 0.6)	No significant difference
Readmission for IE	No significant difference (*p* = 0.63)	No significant difference
Hospital length of stay	Shorter (*p* = 0.03)	Longer hospital stay

**Table 6:** Data comparison.

Variable	PMA Group	Surgery Group	*p*-Value
Number of patients	42	43	
Female patients (%)	73%	73%	
Median age	31 (27–41)	31 (27–41)	
History of IE (%)	86%	86%	
Septic shock on admission (%)	38%	38%	
MRSA infection (%)	38%	38%	
in-hospital mortality	12%	2%	*p* = 0.11
12-month all cause mortality	24%	19%	*p* = 0.57

## Data Availability

The original data presented in this study are publicly available on PubMed; all relevant references are cited in the text and included in the reference list.
